# The Contribution of Proteomics in Understanding Endometrial Protein Expression in Women with Recurrent Implantation Failure

**DOI:** 10.3390/jcm13072145

**Published:** 2024-04-08

**Authors:** Anastasios Potiris, Eleni Alyfanti, Eirini Drakaki, Despoina Mavrogianni, Theodoros Karampitsakos, Pavlos Machairoudias, Spyridon Topis, Athanasios Zikopoulos, Chara Skentou, Periklis Panagopoulos, Peter Drakakis, Sofoklis Stavros

**Affiliations:** 1 Third Department of Obstetrics and Gynecology, Medical School, University General Hospital “ATTIKON”, National and Kapodistrian University of Athens, 12462 Athens, Greece; elenalyfanti@med.uoa.gr (E.A.); theokarampi@med.uoa.gr (T.K.); pavlosmach@med.uoa.gr (P.M.); spyrostopis@med.uoa.gr (S.T.); perpanag@med.uoa.gr (P.P.); pdrakakis@med.uoa.gr (P.D.); sfstavrou@med.uoa.gr (S.S.); 2 First Department of Obstetrics and Gynecology, Medical School, Alexandra Hospital, National and Kapodistrian University of Athens, 11528 Athens, Greece; eirinidrak@med.uoa.gr (E.D.); dmavrogianni@med.uoa.gr (D.M.); 3 Department of Obstetrics and Gynecology, Royal Cornwall Hospital, Treliske, Truro TR1 3LQ, UK; thanzik92@gmail.com; 4 Department of Obstetrics and Gynecology, Medical School of the University of Ioannina, 45110 Ioannina, Greece; haraskentou@uoi.gr

**Keywords:** proteomics, recurrent implantation failure (RIF), endometrial protein expression, biomarkers, endometrial receptivity

## Abstract

Recurrent implantation failure (RIF) poses a significant challenge in assisted reproductive technology (ART) outcomes. The endometrium plays a crucial role in embryo implantation, and its protein expression profile is integral in determining receptivity. Proteomics has emerged as a valuable tool in unraveling the molecular intricacies underlying endometrial receptivity and RIF. The aim of the present review is to analyze the contribution of proteomics to the understanding of endometrial protein expression in women with RIF, based on the results of significant proteomic studies. Medline/Pubmed databases were searched using keywords pertaining to proteomics combined with terms related to RIF. 15 studies were included in the present review. Several proteins have been found to exbibit differential expression in endometrial biopsies and fluid samples between fertile women and women with RIF during the receptive endometrial phase. The profile of endometrial proteins varied significantly among the studies. Nevertheless, similar changes in the expression levels of annexin-6, progesterone receptor, MMP-2, and MMP-9 in the endometrium of women with RIF, were found in more than one study indicating that certain proteins could potentially be effective biomarkers of endometrial receptivity. Proteomics contributes significantly to the understanding of protein expression in the endometrium of women with RIF and the analysis of proteins in endometrial fluid are promising for improving the clinical management of RIF.

## 1. Introduction

Recurrent implantation failure (RIF) is a highly demanding and complex clinical phenomenon that, despite advancements in assisted reproductive technology, remains a major issue affecting approximately 15% of women undergoing in vitro fertilization [1]. Despite the extensive relevant literature, there is still no widely accepted definition. The definition that until recently had the broadest acceptance for RIF is that of Coughlan et al., who define RIF as the failure to achieve clinical pregnancy after the transfer of at least four high-quality embryos following at least three fresh or frozen IVF cycles, in women under the age of 40 [2]. The European Society of Human Reproduction and Embryology (ESHRE) has recently defined RIF as the scenario in which the transfer of embryos considered to be viable has failed to result in a positive pregnancy test sufficiently often in a specific patient to warrant consideration of further investigations and/or interventions [3].

The implantation of the embryo is of utmost importance for the success of IVF and although it is well known that is a very complex process, it is a fact that the precise mechanisms have not yet been fully elucidated. Recurrent implantation failure can be caused by maternal, paternal, and fetal factors, as well as a combination of different factors. Maternal factors include disorders of endometrial receptivity, the immune system, thrombophilia, chronic endometritis and inflammation, endocrine disorders, uterine polyps, and uterine anatomical abnormalities [4,5]; additionally, thin endometrium and additional factors such as maternal age, BMI, smoking, alcohol consumption, and stress. Embryonic factors include various types of chromosomal abnormalities in the embryo, translocations, inversions, deletions, and mosaicism [6,7]. It is important to note that disorders related to endometrial receptivity and biochemical alterations of the dialogue between the endometrium and the blastocyst account for approximately two-thirds of cases of recurrent implantation failure [8,9]. Despite the extensive research and suggestions that have been made for the correct diagnosis, prognosis and various treatment options for RIF, there are still knowledge gaps that need further investigation.

Proteomics, a high-throughput approach enabling the comprehensive analysis of protein expression, post-translational modifications, and protein–protein interactions, has emerged as a powerful tool in reproductive medicine providing exceptionally high-precision qualitative and quantitative results. There are numerous techniques applied in proteomic analysis and can be divided into four categories. These include conventional techniques, used for protein purification and based on chromatography, advanced techniques, quantitative techniques, and high-performance techniques [10]. The use of proteomics offers the ability to reveal the mechanisms of complex biological processes and conditions, including fertilization, embryo implantation, embryo development, and pregnancy [11]. Proteomic analysis has been widely applied in modern research, which aims to gain a deeper understanding of the pathology of recurrent implantation failure and to find reliable biomarkers of endometrial receptivity.

This review aims to summarize the current state of knowledge, regarding the contribution of several proteomic studies carried out in the last two decades, in deciphering endometrial protein expression patterns in women with RIF, with a focus on its potential implications for improving ART outcomes.

## 2. Materials and Methods

To provide a comprehensive review of the literature published up to November 2023, Medline/PubMed databases were searched using keywords pertaining to proteomics, including “proteomic analysis”, “proteome”, “protein expression profiles”, combined with terms related to recurrent implantation failure (RIF) such as “implantation failure”, “endometrial receptivity”, “embryo implantation”, and “endometrial receptivity biomarkers”. These terms were either used separately or in combination with the help of the Boolean administration (OR, AND). All articles published after 2003 and with an English title and abstract were initially accepted. Reviews, systematic reviews, and studies that examined the endometrium of women who, apart from RIF, were diagnosed with other pathological conditions were not included.

In the screening process, the title and abstract of 20 studies were screened independently by two reviewers (A.P. and E.A.). Subsequently, a full-text assessment was performed. If a study was selected only by one reviewer, the decision was taken by a third reviewer (D.M.). 17 studies were selected for full-text assessment and after careful evaluation 15 articles were included in the present review. Data extraction was performed by a single reviewer (E.A.) and validated by a second reviewer (A.P.).

## 3. Results

### 3.1. Comparison of Protein Expression in Endometrial Tissue Samples between Fertile Women and Women with Recurrent Implantation Failure

The study by Brosens et al., published in 2010, demonstrated that changes in the expression, modification, or regulation of apolipoprotein-A1 (apoA-I) by paracrine embryonic signals play a significant role, as they can lead to implantation failure. apoA-I is the major protein component of high-density lipoprotein (HDL) particles and is a key part in lipid metabolism. The research team employed a series of proteomic techniques, such as surface-enhanced laser desorption/ionization time-of-flight mass spectrometry (SELDI-TOF-MS), immunohistochemistry, Western blot analysis, and enzyme-linked immunosorbent assay (ELISA). A comparison was made between endometrial samples during the mid-secretory phase from women with recurrent implantation failure and fertile women. The analysis using SELDI-TOF-MS showed higher levels of apoA-I expression in women with RIF (*p*-value = 0.0002); however, verification via Western blotting did not reveal a significant increase in apoA-I levels in samples from women with RIF compared to fertile controls. To investigate the response of apolipoprotein-A1, which is a potent anti-inflammatory molecule, to embryonic hormonal stimuli, human chorionic gonadotropin (hCG) was injected into the histological samples. The administration of hCG resulted in a strong inhibition of apoA-I expression in differentiated endometrial tissue cultures from the comparison groups [12].

The team of Mariee et al., in 2012 applied the method of immunohistochemistry to compare the levels of interleukin-15 (IL-15) and leukemia inhibitory factor (LIF) in the endometrium of women with recurrent implantation failure (RIF) and in the endometrium of women with normal fertility. Furthermore, they examined the relationship between the levels of IL-15 and LIF with the number of uterine natural killer cells (uNK cells). IL-15 is a pleiotropic cytokine that belongs to the four α-helix bundle family of cytokines and plays an important role in both innate and adaptive immunity. One of its basic functions is the stimulation of Natural Killer (NK) cells. LIF belongs to the interleukin-6 (IL-6) cytokine family and participates in various biological processes, particularly in the regulation of stem cell pluripotency, inflammation, and the immune response, while it has been proven that LIF produced by the endometrial tissue is involved in the crosstalk between the embryo and the uterus during implantation. The results of this study showed that the quantity of LIF in the epithelial tissue of the endometrial glands in women with recurrent implantation failure (RIF) was lower compared to fertile women (*p*-value = 0.01). In contrast, the level of IL-15 in stromal cells of women with RIF was higher than that of the control group (*p*-value = 0.009). Finally, a significant correlation was observed between the number of uNK cells and the expression of IL-15 in the stromal cells (r = 0.427, *p*-value = 0.001), while there was no correlation between the expression of LIF and the number of uNK cells [13].

In 2014, the team of Garrido-Gómez et al. conducted a comparison between endometrial biopsy samples from women undergoing hormone replacement therapy, which had been characterized as receptive and non-receptive based on evaluation with the ERA (Endometrial Receptivity Analysis) test. These samples were taken following administration of progesterone for 5 consecutive days. Protein isolation was performed, followed by differential gel electrophoresis (DIGE) and MALDI-MS analysis, which identified 24 proteins with differential expression between receptive and non-receptive endometrial samples. Subsequently, through in silico analysis, a network was created with these 24 proteins along with various introduced nodes, aiming to determine if any pathway or group of proteins was involved in the differential receptivity status of the endometrium between the two comparison groups. Two statistically significant pathways were identified in comparison between samples from receptive (ERA-R) and non-receptive endometria (ERA-NR): the “carbohydrate biosynthesis pathway” and the “nuclear mRNA splicing through spliceosome pathway”. This network highlighted phosphoglucomutase 1 (PGM1), alpha-enolase (ENO1), and sialic acid synthase (SIAS) as dysregulated proteins associated with carbohydrate metabolism pathways, which may be involved in altering the metabolic state of the endometrium and thereby increasing its receptivity. The results of the DIGE analysis were validated using Western blotting and immunohistochemistry for two proteins: annexin-6 (ANXA6) and progesterone receptor membrane component 1 (PGRMC1). ANXA6 belongs to the annexin family of calcium-dependent phospholipid-binding proteins and is involved in membrane binding, endocytosis, intracellular trafficking, and signaling pathways, including apoptosis signaling pathway. PGRMC1 is a versatile protein involved in steroid hormone signaling, cellular signaling pathways, gene expression regulation, and membrane organization. The results obtained by Western blot and immunohistochemistry confirmed the increased expression of ANXA6 and PGRMC1 in ERA-NR endometrial samples compared to ERA-R samples. Based on these results, the research team proposed ANXA6 and PGRMC1 proteins as new molecular targets and the “carbohydrate biosynthesis pathway” and “nuclear mRNA splicing through the spliceosome” as significant pathways associated with the creation of receptive endometrium [14].

The scientific team of Long et al. investigated in 2016 the relationship between telomerase and the expression of steroid hormone receptors in recurrent implantation failure (RIF). Telomerase is a ribonucleoprotein which adds guanine-rich repetitive sequences to the 3′ end of telomeres, to maintain the length of DNA telomeres. Estrogen receptor alpha (ER alpha) and progesterone receptor (PR) are two nuclear receptors, that are activated by estrogens and progesterone, respectively, influence gene expression and they play an essential role during the implantation window. Endometrial biopsies were collected from patients with RIF and from fertile women. Using the real-time PCR technique, changes were detected at the transcriptional level of the expression of telomerase reverse transcriptase (Tert), estrogen receptor alpha (ER alpha) and progesterone receptor (PR). Subsequently, Western blotting and immunohistochemistry techniques were applied to analyze the expression of TERT and ER alpha at the protein level. The protein expression of TERT was increased in patients with RIF (*p*-value < 0.0001) and was localized in the endometrial epithelium and stromal cells. However, the signal from the ER alpha in the stromal cells of women with RIF was weaker compared to the control samples (*p*-value = 0.0056). In conclusion, this study demonstrated that induction of TERT is significantly correlated with reduced production of ER alpha, which in turn directly affects the protein environment of the endometrium and contributes to embryo implantation failure [15].

The research study by Dhaenes et al. in 2018 investigated the protein profile of in vitro cultured endometrial stromal cells obtained from women with recurrent implantation failure (RIF), recurrent pregnancy loss (RPL) and from fertile women. The study compared the protein profile on the first day (non-decidualized samples) of culture with that on the fifth day (decidualized samples). Proteins were isolated and analyzed using high-resolution mass spectrometry. The results showed an increase in ANXA6 (annexin 6), PSMC5 (26S proteasome regulatory subunit 8), and FSCN1 (fascin) in the RIF group, while PBXIP1 (pre-B-cell leukemia transcription factor-interacting protein 1) decreased during decidualization (5th day of culture) (FDR = 10%, *p*-value < 0.05). PSMC5 is a component of the 26S proteasome complex, which plays a critical role in protein degradation. FSCN1 is a key regulator of actin bundling and is involved in cell movement and invasion, while PBXIP1 plays important roles in cellular processes such as transcriptional regulation and apoptosis. In the RPL group, RPS25 (40S ribosomal protein S25) and ACADVL (very long-chain specific acyl-CoA dehydrogenase, mitochondrial) decreased between non-decidualized and decidualized samples (FDR = 10%, *p*-value < 0.05). ACADVL catalyses the first step of the fatty acid beta-oxidation pathway, which is critical for the decidualization process. In the group of fertile women, the proteins vimentin (VIM) and RPL23A (60S ribosomal protein L23a) exhibited decreased concentration (FDR = 10%, *p*-value < 0.05). After comparing expression ratios between decidualized and non-decidualized samples across all groups, six proteins with differential expression were identified: DUX4L2 (double homeobox protein 4-like protein 2), CNPY4 (protein canopy homologue 4), PDE7A (high affinity cAMP-specific 3′,5′-cyclic phosphodiesterase 7A), CTSK (cathepsin K), PCBP2 (poly(rC)-binding protein 2), and PSMD4 (26S proteasome non-ATPase regulatory subunit 4) (FDR = 10%, *p*-value < 0.05). DUX4L2 is a member of the double homeobox (DUX) family of transcription factors. CNPY4 is a member of the Canopy (CNPY) family of proteins which plays a multifaceted role in maintaining cellular homeostasis by regulating protein folding, the ER stress response, and the secretion of proteins. PDE7A is an enzyme that participates in the regulation of intracellular levels of cyclic adenosine monophosphate (cAMP). CTSK is a cysteine protease enzyme which is involved in collagen degradation and PSMD4 is a subunit of the 19S regulatory particle of the 26S proteasome, which participates in the regulation of protein degradation. PCBP2 is a member of the heterogeneous nuclear ribonucleoprotein (hnRNP) family of RNA-binding proteins and has an important role in post-transcriptional regulation of gene expression. Serotransferrin also showed differential expression in the comparison between the RPL group and fertile women (FDR 3%, *p*-value < 0.01). Changes in the expression levels of serotransferrin, ANXA6, ACDVL, and VIM were further validated using the Western blot method [16].

In 2019, the research team of Bielfeld et al. conducted a proteomic analysis of endometrial tissue obtained through biopsy from patients with recurrent implantation failure (RIF) and from women with proven fertility. Using mass spectrometry, 2120 proteins were identified, three of which exhibited statistically significant changes in their quantity in the endometrial tissue of patients with RIF compared to fertile women. Specifically, the results showed an increase in the quantity of epiplakin-1 (EPPK1) and Bcl-2-associated transcription factor 1 (BCLAF1), and a decrease in the quantity of prothymosin-alpha (PTMA) in women with RIF. EPPK1 is a multifunctional protein crucial in maintaining the structural integrity of epithelial tissues, regulating cell adhesion and migration, and contributing to cytoskeletal dynamics. BCLAF1 plays important roles in various cellular processes, including apoptosis, transcriptional regulation, RNA splicing, and DNA damage response. PTMA is a ubiquitous polypeptide with both intracellular and extracellular functions. Intracellularly, it acts as an anti-apoptotic and proliferation mediator, while extracellularly, serves as a biologic response modifier, influencing immune responses and contributing to immune regulation. Additionally, an analysis of the endometrial tissue was performed following in vivo administration of a human chorionic gonadotropin (hCG) substitute. The endometrial administration of hCG helped increase the expression of intracellular proteins, HIF1 (Hypoxia-Inducible Factor 1) signaling, and chemokine production. Specifically, patients with RIF showed a 19% higher clinical pregnancy rate after endometrial hCG administration prior to embryo transfer (ET), compared to failed attempts in previous cycles. Based on these findings, the research team suggests that endometrial hCG administration before ET may improve chemical communication between the embryo and endometrium, enhance angiogenesis and immune response, thereby resulting in the increase in pregnancy rate in patients with RIF [17].

In the study conducted by Wang et al. and published in 2021, a screening was performed for endometrial proteins with differential expression during the window of implantation (WOI), using the iTRAQ labeling technique in combination with 2D LC-MS/MS, aiming to identify potential biomarkers for patients with recurrent implantation failure (RIF). The endometrial tissue samples were obtained by hysteroscopy during the phase of WOI, from September 2016 to December 2019, and simultaneously during this period, clinical data were collected. Patients were divided into two groups according to pregnancy outcomes: a group of women with reproductive failure (RIF) and a control group of women who achieved successful pregnancy. The results revealed 82 differentially expressed proteins in patients with RIF compared to the control group, including 55 proteins with increased expression (>1.50 fold, *p*-value < 0.05) and 27 proteins with decreased expression (<0.67 fold, *p*-value < 0.05). Bioinformatics analysis using the String analysis software highlighted interactions among these proteins, which were grouped into two categories: ribosomal proteins and homeostatic blood proteins. The most statistically significant enriched biological processes identified by Gene Ontology analysis included the downregulation of hydrolase activity, blood microparticles, and enzyme inhibition activity. Specifically, seven proteins (SPB6, APOA1, GMIP, THBG, CBG, ANT3, and FETUA) involved in hydrolase activity were identified, another seven proteins (VTDB, IGHG4, APOA1, A1AG2, FETUA, ANT3, and A1AG1) were found in the category of blood microparticles, and yet another seven proteins (ANT3, FETUA, CBG, ASPN, SPB6, THBG, and APOA1) were implicated in the inhibition of various enzymes. Additionally, verification was performed for the levels of antithrombin-III (ANT3), corticosteroid-binding globulin (CBG), and fetuin-A (FETUA) in the endometrium using the Western blot method, and significantly higher levels of CBG (1.39-fold, *p*-value = 0.003) and fetuin-A (1.47-fold, *p*-value = 0.002) were observed in patients with RIF. CBG is a glycoprotein whose main function is to bind glucocorticoids and progesterone in the bloodstream, thereby regulating their distribution and availability to target tissues throughout the body. FETUA is a multifunctional glycoprotein involved in various physiological processes including calcification inhibition, regulation of insulin sensitivity, modulation of inflammation, lipid metabolism, and bone mineralization. Based on the above results, the research team proposed corticosteroid-binding globulin (CBG) and fetuin-A as biomarkers for recurrent implantation failure [18].

The study by Fu et al. in 2022 demonstrated that aberrant expression of DNA topoisomerase-alpha (TOP2A), which is a critical enzyme involved in DNA replication, transcription, and repair, affects endometrial decidualization and alters the timing of the “window of implantation” thereby leading to implantation failure. The research team conducted proteomic analysis to assess differentially expressed proteins between endometrial tissue samples of patients with RIF and fertile women. The expression patterns of TOP2A in the pre-implantation endometrium of patients with RIF were determined using immunohistochemistry, Western blotting, and qRT-PCR. Furthermore, T-HESCs (Immortal human ESCs) were generated with TOP2A gene silencing (sh-TOP2A) using lentiviral vectors and the expression of TOP2A in T-HESCs was manipulated to investigate its role in the process of decidualization. The TOP2A-related changes in decidualization were examined using mRNA sequencing and subsequently validated by Western blotting and immunofluorescence. The results showed that TOP2A is widely expressed in both stromal and glandular epithelial cells of the endometrium, and its expression appeared to be significantly lower in the mid-secretory phase of the endometrium in women with RIF (*p*-value < 0.01). TOP2A expression was downregulated under stimulation by 8-bromo-cAMP and MPA [19].

In 2023, the research study by Yang et al. proposed that the levels of the proteins TPPP3 (tubulin polymerization-promoting protein family member 3), S100A13 (S100 Calcium Binding Protein A13), HSD17B2 (17b-hydroxysteroid dehydrogenase 2), and AZGP1 (alpha-2-glycoprotein 1, zinc binding) in the endometrium can be used as markers for endometrial receptivity and the diagnosis of women with recurrent implantation failure (RIF). TPPP3 is a member of the tubulin polymerization promoting protein family, involved in regulating microtubule dynamics within cells. S100A13 is a small calcium-binding protein belonging to the S100 family, which plays important roles in a series of cellular processes, such as calcium homeostasis, cell proliferation, apoptosis, and inflammation. HSD17B is an enzyme essential for the synthesis and metabolism of estrogens and androgens. AZGP1 is a secretory adipose factor which participates in metabolic processes such as lipolysis and glucose transport, acts as a tumor suppressor and may be involved in immune modulation. The research team compared endometrial tissues obtained from women with RIF to those from women with normal fertility, taken 7–9 days after the peak of luteinizing hormone (LH). Using the iTRAQ technique, 2063 proteins with differential expression were identified, which are involved in various biological processes such as protein translation, mitochondrial function, oxidoreductase activity, amino acid, and fatty acid metabolism. Ultimately, after bioinformatics analysis, the proteins TPPP3, S100A13, HSD17B2, and AZGP1 were identified as potential biomarkers of receptive endometrium and further validation was performed using Western blot and immunohistochemistry. Specifically, the iTRAQ analysis revealed that the expression of the proteins HSD17B2 and AZGP1 decreased in samples from patients with RIF compared to those of the control group, while the proteins TPPP3, APEX1, and S100A13 showed increased expression in the RIF group. The results from Western blot and immunohistochemistry showed that the levels of TPPP3, HSD17B2, S100A13, and AZGP1 were significantly reduced in the endometrium of patients with RIF [20].

Another study published in 2023 by Zhao et al. compared the protein expression in endometrial tissues collected from women with recurrent implantation failure (RIF) and from women with recurrent implantation successes (RIS). The differential expression of proteins between individuals with RIF and those with RIS was identified using the iTRAQ and LC-MS/MS techniques. Overall, 35,071 peptides and 6290 proteins were identified in the samples of both comparison groups using the iTRAQ method. According to the criteria for defining differentially expressed proteins set by the research team (fold change > 1.3 or <0.77 and *p*-value < 0.05), 425 proteins with increased expression and 200 proteins with decreased expression were found in the RIF group compared to the RIS group. The bioinformatics analysis (GO enrichment and KEGG pathway analysis) revealed that these proteins were enriched in biological processes related to Wnt and TGF-β signaling, smooth muscle contraction, ubiquitin-mediated proteolysis, and tight junctions. To confirm the above results for the proteins that showed differential expression, validation was performed using qPCR and Western blotting. It was revealed that claudin-4, p38 kinase, AMP-activated protein kinase subunit beta (PRKAB), and NF-kB inhibitor subunit-beta (IKB-b) had increased expression, while myosin-2 and protein kinase G (PKG2) had significantly decreased expression in women with RIF [21]. Claudin-4 is a tight junction protein which plays a crucial role in maintaining the barrier function of tight junctions and regulates paracellular permeability. p38 kinase is a versatile signaling molecule which is involved in stress responses, inflammation, cell cycle regulation, apoptosis, differentiation, response to growth factors and tissue regeneration. PRKAB is a component of the AMPK complex, which acts as a cellular energy sensor and regulator of metabolic pathways, influencing cell growth and autophagy. IKB-b is a critical regulator of the NF-kB signaling pathway and its roles include regulating the inflammatory response, cell survival, development, and immune response. Myosin-2 is a versatile motor protein involved in muscle contraction, cell movement, cell adhesion and communication. PKG2 is involved in cGMP signaling pathways and plays roles in smooth muscle relaxation, cardiovascular function, platelet function, neuronal function, and the regulation of ion channels, cell cytoplasm and cytoskeleton array. However, contrary to the findings of this study, in the study by Pérez-Debén et al., where iTRAQ technology was also applied and a comparison was made between endometrial samples from fertile women, women with an intrauterine contraceptive device, and women with a history of recurrent implantation failure, no differences were found in the protein composition of the endometrium between patients with RIF and fertile women [22].

### 3.2. Comparison of Protein Expression between Endometrial Fluid Samples from Fertile Women and Women with Recurrent Implantation Failure

To investigate noninvasive methods for assessing endometrial receptivity, some studies have examined the different protein composition of endometrial fluid samples. In 2003, the team of Inagaki et al. used the method of zymography to compare the levels of interleukin-1beta (IL-1beta), tumor necrosis factor-alpha (TNF-alpha), interferon-gamma (IFN-gamma), leukemia inhibitory factor (LIF), interleukin-10 (IL-10), and the activity of matrix metalloproteinases 2 and 9 (MMP-2 and MMP-9) among samples of intrauterine fluid obtained from fertile women and patients with recurrent implantation failure (RIF). IL-1beta and TNF-alpha are pro-inflammatory cytokines that play a key role in the promotion of inflammation and immune responses. IFN-gamma is a potent cytokine which activates macrophages, enhances the activity of NK cells and cytotoxic T cells, and has antiviral effects. IL-10 is an anti-inflammatory cytokine that facilitates the regulation of the immune response and maintains immune balance, through suppression of pro-inflammatory cytokines and immune cells. MMP-2 and MMP-9, both members of the gelatinase subgroup of MMPs, are involved in tissue remodeling, ECM degradation, and various pathological processes. It was observed that the activity levels of MMP-2, MMP-9, and IL-1beta were increased in women RIF compared to fertile women, while the concentrations of IFN-gamma and IL-10 were significantly lower (*p*-value = 0.05). Regarding TNF-alpha, there was a trend towards increased expression in samples from women with RIF, but this difference was not statistically significant. Similarly, the expression of LIF did not show a statistically significant difference between the compared groups [23]. The higher levels of MMP-2 and MMP-9 in women with RIF were also confirmed by the study of Yoshii et al. in 2013 [24]. The research team indicated that this increase reflected an inflammatory state in the endometrium, and therefore, they proceeded with treatment using antibiotics and corticosteroids aiming to reduce inflammation in women with RIF. This treatment likely improved the endometrial environment by limiting the activity of matrix metalloproteinases (MMPs), and consequently led to significantly better pregnancy outcomes in the group of patients who underwent this therapy [24,25].

The study by Azkargorta et al., published in 2018, confirmed by large-scale proteomic techniques that the endometrial fluid exhibits a different protein composition between women who achieve successful embryo implantation in IVF cycles and those who experience implantation failure. The collection of endometrial fluid was performed immediately before embryo transfer. The results of the proteomic and bioinformatic analysis revealed that samples from women experiencing implantation failure exhibited significant dysregulation in processes controlling endometrial receptivity, such as antimicrobial response, cellular interaction, immune response and signaling during inflammation. Also, findings of this study indicate a scenario where the ability to respond to oxidative stress is compromised in non-receptive endometrial fluid aspirate (EFA). This is partly due to the potential inhibition of Nrf2, which is a key regulator of the oxidative stress response, as it is responsible for activating a range of antioxidant genes. Overall, eight proteins were identified with strong dysregulation: Mucin-16 (MUC16), Heat shock 70 kDa protein 4 (HSP74), Prostaglandin E synthase 3 (TEBP), Follistatin-related protein 3 (FSTL3), Glycogen phosphorylase (PYGB), Ubiquitin-like modifier-activating enzyme 1 (UBA1), Peptidyl-prolyl cis-trans isomerase A (PPIA), and 14-3-3 protein epsilon (1433E) (q < 0.05). One of them, glycogen phosphorylase B (PYGB), was selected for confirmatory analysis by Western blot, where its reduced expression in samples from women with RIF was verified (*p*-value < 0.05). PYGB is an enzyme that plays a key role in glycogen metabolism and dysregulation of PYGB can lead to glycogen storage diseases. MUC16 provides a protective, lubricating barrier against particles and infectious agents at mucosal surfaces, as well as suppresses the cytolytic responses of NK cells. HSP74 is a member of the HSP70 family, functioning as a molecular chaperone to assist in protein folding and refolding under stress conditions, while it also plays a crucial role in maintaining protein homeostasis. TEBP has anti-inflammatory and immunomodulatory effects. FSTL3 is a multifunctional protein involved in various biological processes, including cell development, inflammation regulation, and metabolism. UBA1 is an E1 enzyme in the ubiquitin-proteasome system (UPS), which is responsible for tagging proteins with ubiquitin for degradation. PPIA, also known as cyclophilin A, is an enzyme that catalyses protein folding, and plays a role in the regulation of the immune response and protein cycle. 1433E is a member of the 14-3-3 protein family, which binds to various target proteins to modulate their activity and is involved in the regulation of ion concentration, cell–cell adhesion and cellular signaling. Based on the results of this study, the research team supported that the analysis of endometrial fluid has the ability to detect the increased inflammatory status in non-receptive endometrial samples. Additionally, they proposed PYGB as a potential biomarker for predicting endometrial receptivity or the success of implantation [26].

The team of Kasvandik et al. in 2019, analyzed endometrial fluid samples (UF) obtained from the endometrium of early and mid-secretory phase of fertile women and women with RIF, to determine if the proteome of uterine fluid could be used to monitor the window of implantation (WOI). Using mass spectrometry, 367 proteins were identified, which underwent significant changes during the transition from the early to mid-secretory phase of the endometrium (q ≤ 0.05, fold change (FC) range: −15.2 to +60.9). Subsequently, 45 of these proteins (with |FC| ≥ 5, q < 0.05) were further analyzed by targeted MS, and 21 were found to exhibit similar levels between the early secretory phase group of fertile women and the mid-secretory phase group of women with RIF, indicating the temporal shift of the window of implantation in women with RIF. The panel of proteins PGR (progesterone receptor), NNMT (nicotinamide N-methyltransferase), SLC26A2 (Solute Carrier family 26 member 2), and LCN2 (lipocalin-2) showed high specificity (91.7%) and sensitivity (91.7%) in distinguishing samples from the mid-secretory phase from those of the early secretory phase. The same panel distinguished with high specificity (91.7%) and sensitivity (96.6%) the samples from the mid-secretory phase of the endometrium of fertile women from those of women with RIF. PGR is a nuclear receptor protein that mediates the biological effects of progesterone hormone. NNMT is an enzyme involved in the metabolism of nicotinamide, as well as in the regulation of cellular methylation and metabolism. SLC26A2 is a sulfate transporter protein involved in the transport of sulfate ions across cell membranes. LCN is an iron-trafficking protein participating in multiple processes such as apoptosis and innate immunity. Based on the above results, this study demonstrated that the proteins present in uterine fluid, obtained with minimal invasiveness, can be used to assess endometrial receptivity. Furthermore, women with RIF appear to have a differentiated protein profile during the mid-secretory phase of the endometrium, a factor that is highly likely to contribute to the low success rate of implantation [27].

Table 1 summarizes the proteins that showed differential expression in the endometrium of women with recurrent implantation failure (RIF) and is based on the results of all the studies mentioned in this review, both those that compared protein expression in endometrial biopsies and those that analyzed endometrial fluid samples.

## 4. Discussion

Recurrent implantation failure (RIF) undeniably constitutes a serious and multifaceted problem in assisted reproduction, for which significant research effort has been made in recent years by many scientific teams. The aim of the research studies that have been carried out to date, which, for the most part, included genetic and transcriptomic studies, has been to understand the causes and mechanisms that can lead to RIF, to develop specific tests for the early diagnosis of women prone to implantation failure and to find new therapeutic approaches.

Over the last two decades, many potential molecular markers and proteins associated with endometrial receptivity have been identified. Despite all efforts, current tests for endometrial receptivity prior to embryo transfer, which involve invasive biopsies and rely on endometrial transcriptome analysis, result in modest improvements in implantation outcomes. According to the results of a meta-analysis, the rates of ongoing pregnancy (OPR) and live births (LBR) were comparable between patients with RIF diagnosed after endometrial receptivity testing, with non-receptive endometrium, who underwent personalized embryo transfer (p-ET), and patients with receptive endometrium who underwent embryo transfer based on the standard protocol [28]. On the contrary, according to a recent multicenter cohort study in patients with a previous failed embryo transfer, the LBR and cumulative LBR were higher after embryo transfer based on the standard protocol compared to personalized embryo transfer (p-ET), which was performed after endometrial receptivity testing [29]. ESHRE supports that there is insufficient evidence to recommend the use of currently available endometrial receptivity tests in ART and states that more studies are needed to determine their value in managing patients with RIF [3]. Therefore, the field of human reproductive medicine needs more precise and quantitative, yet rapid and non-invasive assessments to further improve pregnancy rates.

Proteomics represents a modern, technology-driven science that studies proteins and their post-translational modifications and interactions, providing exceptionally high-precision qualitative and quantitative results. The proteome is more than the translated products of the genetic material, as proteins significantly differ in stability and rates of modification, have different spatiotemporal regulation, undergo epigenetic and post-translational modifications, and interact with other proteins to create structural and functional complexes [30]. Therefore, the proteomic profile is simultaneously multifaceted and dynamic, spatially and temporally, and proteomic analysis is capable of reflecting various phases of cell differentiation and states.

In transcriptomic studies, compelling evidence has shown that there are distinct transcriptional characteristics in the endometrium during various phases [31,32], and a recent test for endometrial receptivity (ERA) has been applied as a diagnostic tool in some assisted reproduction centers [33,34]. However, there are still some limitations in the depth of understanding of endometrial receptivity based on transcriptional results because not all RNA messages are translated into proteins, and post-transcriptional, translational, and post-translational regulations are extensively applied. The use of proteomic analysis in the investigation of endometrial receptivity is extremely useful and promising with the new innovative methods that it includes, as it can provide more information and contribute both in understanding the protein expression of the endometrium during the “window of implantation”, as well as in finding reliable biomarkers for assessment of the receptivity of the endometrium, with the ultimate goal of timely and accurate therapeutic intervention in the cases of women with RIF.

Big data analysis and proteomics, the large-scale study of proteins, have become increasingly intertwined in modern biological research. One of the proteomic techniques that is widely used for protein identification and quantification is Mass spectrometry (MS). MS is a rapid and reliable method for proteomic analysis, especially when coupled with liquid chromatography (LC-MS/MS) for high-throughput applications. Additionally, with the appropriate setup, MS is able to provide simultaneous results for a large number and types of samples [35]. Multiplexed assays, such as immunoassays or protein arrays, can also be relatively fast depending on the specific technology and allow the simultaneous measurement of multiple proteins in a single sample, providing high throughput. They can also provide simultaneous results for a large number of samples, especially in the case of microarray-based methods and are preferred for targeted analysis of specific proteins or pathways [36].

In the present review, several important proteomic studies of the endometrium conducted over the last 20 years were discussed. Studies comparing endometrial tissues of fertile women with those of women with RIF mostly showed that there are proteins exhibiting differential expression between the compared groups during the receptive endometrial phase. Regarding studies focusing on protein expression in the endometrial fluid, they also demonstrate a differentiated proteomic profile during the mid-secretory phase of the endometrium. Overall, several proteins considered to be associated with endometrial receptivity have been identified in 14 of 15 studies that are concluded in this review, in both samples of endometrial tissue and endometrial fluid. However, the number and profile of proteins in different phases of the endometrium varied significantly among studies. These variations are most likely due to the different experimental conditions of studies. Nevertheless, it could be proposed that the proteins revealed to be important for endometrial receptivity and are common in the majority of studies may after further research serve as effective biomarkers. Such proteins are annexin-6 [14,16], progesterone receptor [14,27], MMP-2 and MMP-9 [23,24].

However, while many biomarkers have been proposed for endometrial receptivity, it is necessary for the optimal set of them to be selected and validated in multicenter, prospective, and reliable randomized trials to establish their clinical application in assisted reproduction. Additionally, due to the many discrepancies among studies, it is necessary to have common designs of proteomic and bioinformatic analysis experiments [11]. The publication by Altmäe et al. has proposed a guide for study design, sample collection and processing, data processing and analysis, as well as the validation of endometrial proteomic studies [37]. Furthermore, although many proteins with differential expression have been identified as correlated with endometrial receptivity through proteomics, achieving synchronization between the embryo and the endometrium is extremely important yet complex as a process. For this reason, the design of in vitro studies, which conduct simultaneous analysis of proteins produced by embryonic trophoblast cells and proteins produced by the endometrium, could be helpful.

## 5. Conclusions

In conclusion, the use of proteomics contributes towards understanding the protein expression of the endometrium in women with RIF and towards finding biomarkers of endometrial receptivity. To our knowledge this is the first review that aims to summarize the differentially expressed proteins in tissue and endometrial fluid in women with recurrent implantation failure. However, there is a need for a standardized model for designing proteomic and bioinformatics analysis experiments. Furthermore, a future goal is the more extensive analysis of proteins in endometrial fluid—which does not require invasive methods—and the implementation of new diagnostic tests using endometrial receptivity biomarkers in clinical practice, leading to personalized embryo transfer, after confirming the synchronization of embryo and endometrial development. It is also necessary to select the optimal set of proposed biomarkers and validate them in multicenter, prospective, and randomized controlled trials, to establish their application in clinical routine. The field of proteomics has strong potential to improve the embryo implantation rate after IVF in women with recurrent implantation failure (RIF), resulting in significantly increased pregnancy and birth rates.

## Figures and Tables

**Table 1:** Summary of proteins with differential expression in the endometrium of women with recurrent implantation failure (RIF).

Protein	Basic Role/Function	Compared Groups	Specimen/Phase of the Cycle	Altered Expression in Women with RIF	Study
Apolipoprotein-A1 (apoA-I)	Lipid metabolism	RIF samples vs. Control	Endometrial biopsies/Follicular phase	Increased expression	Brosens et al. [12]
Interleukin-15 (IL-15)	NK cells proliferation	RIF samples vs. Control	Endometrial biopsies/Ovulation	Increased expression	Mariee et al. [13]
Leukemic inhibitory factor (LIF)	Immune tolerance between endometrial–blastocyst junction	RIF samples vs. Control	Endometrial biopsies/Ovulation	Decreased expression	Mariee et al. [13]
Phosphoglucomutase 1 (PGM1)	Carbohydrate metabolism	ERA-NR samples vs. ERA-R	Endometrial sample/Prereceptive	Decreased expression	Garrido-Gόmez et al. [14]
Alpha-enolase (ENO1)	Carbohydrate metabolism	ERA-NR samples vs. ERA-R	Endometrial sample/Prereceptive	Increased expression	Garrido-Gόmez et al. [14]
Sialic acid synthase (SIAS)	Carbohydrate metabolism	ERA-NR samples vs. ERA-R	Endometrial sample/Prereceptive	Decreased expression	Garrido-Gόmez et al. [14]
Annexin-6 (ANXA6)	Mediates calcium-dependent secretion of exosomes during plasma membrane repair/Involvement in apoptosis mechanism	ERA-NR samples vs. ERA-R	Endometrial sample/Prereceptive	Increased expression	Garrido-Gόmez et al. [14]
		RIF samples vs. Control	Cultured endometrial stromal cells/Secretory	Increased expression	Dhaenes et al. [16]
Progesterone receptor membrane component 1 (PGRMC1)	Progesterone binding/Heme homeostasis/Interaction with cytochromes P450 (CYPs)	ERA-NR samples vs. ERA-R	Endometrial sample/Prereceptive	Increased expression	Garrido-Gόmez et al. [14]
Telomerase reverse transcriptase (TERT)	Creation of telomeres in DNA	RIF samples vs. Control	Endometrial biopsies/Implantation window	Increased expression	Long et al. [15]
Estrogen receptor alpha (ER alpha)	Estrogen binding/DNA binding/Transcription activation	RIF samples vs. Control	Endometrial biopsies/Implantation window	Decreased expression	Long et al. [15]
26S proteasome regulatory subunit 8 (PSMC5)	Protein degradation	RIF samples vs. Control	Cultured endometrial stromal cells/Secretory	Increased expression	Dhaenes et al. [16]
Fascin-1 (FSCN1)	Participation in the organization of the cellular cytoskeleton (formation of microfilaments)	RIF samples vs. Control	Cultured endometrial stromal cells/Secretory	Increased expression	Dhaenes et al. [16]
pre-B-cell leukaemia transcription factor-interacting protein 1 (PBXIP1)	Regulation of protein degradation and autophagy	RIF samples vs. Control	Cultured endometrial stromal cells/Secretory	Decreased expression	Dhaenes et al. [16]
double homeobox protein 4-like protein 2 (DUX4L2)	Regulation of gene expression	RIF non decidualized samples vs. RIF decidualized	Cultured endometrial stromal cells/Secretory	Decreased expression	Dhaenes et al. [16]
protein canopy homologue 4 (CNPY4)	Participation in cell adhesion and cell migration/Signaling/Maintenance of tissue homeostasis	RIF non decidualized samples vs. RIF decidualized	Cultured endometrial stromal cells/Secretory	Increased expression	Dhaenes et al. [16]
high affinity cAMP-specific 3′,5′-cyclic phosphodiesterase 7A (PDE7A)	Involved in the regulation of cyclic adenosine monophosphate (cAMP)	RIF non decidualized samples vs. RIF decidualized	Cultured endometrial stromal cells/Secretory	Decreased expression	Dhaenes et al. [16]
Cathepsin Κ (CTSK)	Involved in collagen degradation	RIF non decidualized samples vs. RIF decidualized	Cultured endometrial stromal cells/Secretory	Decreased expression	Dhaenes et al. [16]
poly(rC)-binding protein 2 (PCBP2)	Regulation of translation/Regulation of gene expression/Participation in intracellular signaling	RIF non decidualized samples vs. RIF decidualized	Cultured endometrial stromal cells/Secretory	Decreased expression	Dhaenes et al. [16]
26S proteasome non-ATPase regulatory subunit 4 (PSMD4)	Regulation of protein degradation	RIF non decidualized samples vs. RIF decidualized	Cultured endometrial stromal cells/Secretory	Decreased expression	Dhaenes et al. [16]
Epiplakin-1 (EPPK1)	Structural support of the epidermis/Immunological response to HPV infection	RIF samples vs. Control	Endometrial scratch biopsies/Luteal Phase	Increased expression	Bielfeld et al. [17]
Bcl-2-related transcription factor (BCLAF1)	Regulation of apoptosis/Regulation of gene transcription/Regulation of immune response	RIF samples vs. Control	Endometrial scratch biopsies/Luteal Phase	Increased expression	Bielfeld et al. [17]
Prothymosin-α (PTMA)	Intracellularly participates in the regulation of the cell cycle, transcription and apoptosis/Extracellularly acts as an immunomodulator	RIF samples vs. Control	Endometrial scratch biopsies/Luteal Phase	Decreased expression	Bielfeld et al. [17]
Corticosteroid-binding globulin (CBG)	Transport and regulation of corticosteroid hormone availability	RIF samples vs. Control	Endometrial biopsies	Increased expression	Wang et al. [18]
Fetuin-A (FETUA)	Involved in inflammatory response/Participation in calcium storage in tissues/Regulation of blood sugar	RIF samples vs. Control	Endometrial biopsies	Increased expression	Wang et al. [18]
DNA topoisomerase-alpha (TOP2A)	Involvement in DNA replication, splicing and repression	RIF samples vs. Control	Endometrial biopsies/Luteal Phase	Decreased expression	Fu et al. [19]
tubulin polymerization-promoting protein family member 3 (TPPP3)	Promotion of microtubule accumulation and maintenance of microtubule system stability	RIF samples vs. Control	Endometrial tissue/Luteal Phase	Decreased expression	Yang et al. [20]
S100 Calcium Binding Protein A13 (S100A13)	Calcium homeostasis/ Cell proliferation/ Apoptosis/Inflammation Response	RIF samples vs. Control	Endometrial tissue/Luteal Phase	Decreased expression	Yang et al. [20]
17b-hydroxysteroid dehydrogenase 2 (HSD17B2)	Synthesis and inactivation of estrogens and androgens	RIF samples vs. Control	Endometrial tissue/Luteal Phase	Decreased expression	Yang et al. [20]
alpha-2-glycoprotein 1, zinc binding (AZGP1)	Lipolysis/Glucose transport	RIF samples vs. Control	Endometrial tissue/Luteal Phase	Decreased expression	Yang et al. [20]
Claudin-4	Formation of tight junction between cells/Control of permeability of substances in epithelium	RIF samples vs. RIS samples	Endometrial biopsy/Luteal Phase	Increased expression	Zhao et al. [21]
Kinase p38	Involvement in stress and inflammatory response/Regulation of cell growth and apoptosis	RIF samples vs. RIS samples	Endometrial biopsy/Luteal Phase	Increased expression	Zhao et al. [21]
AMP-activated protein kinase subunit B (PRKAB)	Regulation of cellular energy balance	RIF samples vs. RIS samples	Endometrial biopsy/Luteal Phase	Increased expression	Zhao et al. [21]
Inhibitor of NF-kB subunit-b (IKB-b)	Regulation of inflammation and immune cellular response/Maintenance of cellular homeostasis	RIF samples vs. RIS samples	Endometrial biopsy/Luteal Phase	Increased expression	Zhao et al. [21]
Myosin-2	Formation of smooth muscle fiber arrays/Participation in movement and the mechanism of tightening the cell membrane	RIF samples vs. RIS samples	Endometrial biopsy/Luteal Phase	Decreased expression	Zhao et al. [21]
Protein Kinase G (PKG2)	Regulation of cell signaling/Regulation of the cell cytoplasm and cytoskeleton array	RIF samples vs. RIS samples	Endometrial biopsy/Luteal Phase	Decreased expression	Zhao et al. [21]
Matrix metalloproteinase-2 (MMP-2)	Degradation of collagen type IV/Participation in angiogenesis/Migration and spreading of cells	RIF samples vs. Control	Endometrial fluid/Luteal Phase	Increased expression	Inagaki et al. [23], Yoshii et al. [24]
Matrix metalloproteinase-9 (MMP-9)	Degradation of collagen and gelatin/Participation in tissue development and regeneration/Involvement in immune response	RIF samples vs. Control	Endometrial fluid/Luteal Phase	Increased expression	Inagaki et al. [23], Yoshii et al. [24]
Interleukin-1beta (IL-1beta)	Promotion of inflammatory and immune response	RIF samples vs. Control	Endometrial fluid/Luteal Phase	Increased expression	Inagaki et al. [23]
Interferon-gamma (IFN-gamma)	Regulation of lymphocyte activity/Control of inflammatory response	RIF samples vs. Control	Endometrial fluid/Luteal Phase	Decreased expression	Inagaki et al. [23]
Interleukin-10 (IL-10)	Suppression of the immune response/Anti-inflammatory action	RIF samples vs. Control	Endometrial fluid/Luteal Phase	Decreased expression	Inagaki et al. [23]
Glycogen phosphorylase B (PYGB)	Regulation of glycogen metabolism	RIF samples vs. Control	Endometrial fluid aspiration/Luteal Phase—Before Embryotransfer	Decreased expression	Azkargorta et al. [26]
Prostaglandin E synthase 3 (TEBP)	Anti-inflammatory and immunomodulatory effects	RIF samples vs. Control	Endometrial fluid aspiration/Luteal Phase—Before Embryotransfer	Decreased expression	Azkargorta et al. [26]
Heat shock 70 kDa protein 4 (HSP74)	Participation in cellular stress control mechanisms/Antigen presentation/Involvement in protein cycle regulation	RIF samples vs. Control	Endometrial fluid aspiration/Luteal Phase—Before Embryotransfer	Decreased expression	Azkargorta et al. [26]
Mucin-16	Regulation of immune responses	RIF samples vs. Control	Endometrial fluid aspiration/Luteal Phase—Before Embryotransfer	Increased expression	Azkargorta et al. [26]
Peptidyl-prolyl cis-trans isomerase A (PPIA)	Protein folding/Regulation of immune response and protein cycle	RIF samples vs. Control	Endometrial fluid aspiration/Luteal Phase—Before Embryotransfer	Decreased expression	Azkargorta et al. [26]
Follistatin-related protein 3 (FSTL3)	Participation in the differentiation of primitive hematopoietic cells/Regulation of metabolism/Possible influence on oocyte maturation	RIF samples vs. Control	Endometrial fluid aspiration/Luteal Phase—Before Embryotransfer	Increased expression	Azkargorta et al. [26]
14-3-3protein epsilon (1433E)	Regulation of ion concentration/Regulation of cell–cell adhesion/Regulation of cellular signaling	RIF samples vs. Control	Endometrial fluid aspiration/Luteal Phase—Before Embryotransfer	Decreased expression	Azkargorta et al. [26]
Ubiquitin-like modifier-activating enzyme 1 (UBA1)	Regulation of protein degradation	RIF samples vs. Control	Endometrial fluid aspiration/Luteal Phase—Before Embryotransfer	Decreased expression	Azkargorta et al. [26]
Progesterone receptor (PGR)	Regulation of menstrual cycle/Promotion of the secretory phase of the endometrium	RIF samples vs. Control	Endometrial fluid/Secretory Phase	Increased expression	Kasvandik et al. [27]
Nicotinamide N-methyltransferase (NNMT)	Methylation of nicotinamide/Regulation of metabolism/Epigenetic regulation/Participation in cell proliferation and differentiation/Participation in inflammatory response	RIF samples vs. Control	Endometrial fluid/Secretory Phase	Decreased expression	Kasvandik et al. [27]
Solute Carrier family 26 member 2 (SLC26A2)	Transport of ions and inorganic molecules/Regulation of ion balance	RIF samples vs. Control	Endometrial fluid/Secretory Phase	Decreased expression	Kasvandik et al. [27]
Lipocalin-2 (LCN2)	Antimicrobial action/Iron binding	RIF samples vs. Control	Endometrial fluid/Secretory Phase	Decreased expression	Kasvandik et al. [27]

RIF: recurrent implantation failure, ERA-NR: endometrial receptivity analysis—non-receptive, ERA-R: endometrial receptivity analysis—receptive, RIS: recurrent implantation successes.

## Data Availability

Not applicable.

## References

[1] Busnelli A., Reschini M., Cardellicchio L., Vegetti W., Somigliana E., Vercellini P. (2020). How common is real repeated implantation failure? An indirect estimate of the prevalence. Reprod. Biomed. Online.

[2] Coughlan C., Ledger W., Wang Q., Liu F., Demirol A., Gurgan T., Cutting R., Ong K., Sallam H., Li T.C. (2014). Recurrent implantation failure: Definition and management. Reprod. Biomed. Online.

[3] Cimadomo D., de Los Santos M.J., Griesinger G., Lainas G., Le Clef N., McLernon D.J., Montjean D., Toth B., Vermeulen N., Macklon N. (2023). ESHRE good practice recommendations on recurrent implantation failure. Hum. Reprod. Open.

[4] Vaduva C.C., Constantinescu C., Serbanescu M., Dara L., Oancea M.D., Carp-Veliscu A. (2023). The association between endometrial polyps, chronic endometritis, and IVF outcomes. Eur. Rev. Med. Pharmacol. Sci..

[5] Buzzaccarini G., Vitagliano A., Andrisani A., Santarsiero C.M., Cicinelli R., Nardelli C., Ambrosini G., Cicinelli E. (2020). Chronic endometritis and altered embryo implantation: A unified pathophysiological theory from a literature systematic review. J. Assist. Reprod. Genet..

[6] Panagopoulos P., Mavrogianni D., Christodoulaki C., Drakaki E., Chrelias G., Panagiotopoulos D., Potiris A., Drakakis P., Stavros S. (2023). Effects of endocrine disrupting compounds on female fertility. Best Pract. Res. Clin. Obstet. Gynaecol..

[7] Ma J., Gao W., Li D. (2022). Recurrent implantation failure: A comprehensive summary from etiology to treatment. Front. Endocrinol..

[8] Potiris A., Perros P., Drakaki E., Mavrogianni D., Machairiotis N., Sfakianakis A., Karampitsakos T., Vrachnis D., Antonakopoulos N., Panagopoulos P. (2024). Investigating the Association of Assisted Reproduction Techniques and Adverse Perinatal Outcomes. J. Clin. Med..

[9] Craciunas L., Gallos I., Chu J., Bourne T., Quenby S., Brosens J.J., Coomarasamy A. (2019). Conventional and modern markers of endometrial receptivity: A systematic review and meta-analysis. Hum. Reprod. Update.

[10] Aslam B., Basit M., Nisar M.A., Khurshid M., Rasool M.H. (2017). Proteomics: Technologies and Their Applications. J. Chromatogr. Sci..

[11] Hernandez-Vargas P., Munoz M., Dominguez F. (2020). Identifying biomarkers for predicting successful embryo implantation: Applying single to multi-OMICs to improve reproductive outcomes. Hum. Reprod. Update.

[12] Brosens J.J., Hodgetts A., Feroze-Zaidi F., Sherwin J.R., Fusi L., Salker M.S., Higham J., Rose G.L., Kajihara T., Young S.L. (2010). Proteomic analysis of endometrium from fertile and infertile patients suggests a role for apolipoprotein A-I in embryo implantation failure and endometriosis. Mol. Hum. Reprod..

[13] Mariee N., Li T.C., Laird S.M. (2012). Expression of leukaemia inhibitory factor and interleukin 15 in endometrium of women with recurrent implantation failure after IVF; correlation with the number of endometrial natural killer cells. Hum. Reprod..

[14] Garrido-Gomez T., Quinonero A., Antunez O., Diaz-Gimeno P., Bellver J., Simon C., Dominguez F. (2014). Deciphering the proteomic signature of human endometrial receptivity. Hum. Reprod..

[15] Long N., Liu N., Liu X.L., Li J., Cai B.Y., Cai X. (2016). Endometrial expression of telomerase, progesterone, and estrogen receptors during the implantation window in patients with recurrent implantation failure. Genet. Mol. Res..

[16] Dhaenens L., Lierman S., De Clerck L., Govaert E., Deforce D., Tilleman K., De Sutter P. (2019). Endometrial stromal cell proteome mapping in repeated implantation failure and recurrent pregnancy loss cases and fertile women. Reprod. Biomed. Online.

[17] Bielfeld A.P., Pour S.J., Poschmann G., Stuhler K., Krussel J.S., Baston-Bust D.M. (2019). A Proteome Approach Reveals Differences between Fertile Women and Patients with Repeated Implantation Failure on Endometrial Level(-) Does hCG Render the Endometrium of RIF Patients?. Int. J. Mol. Sci..

[18] Wang C., Feng Y., Zhou W.J., Cheng Z.J., Jiang M.Y., Zhou Y., Fei X.Y. (2021). Screening and identification of endometrial proteins as novel potential biomarkers for repeated implantation failure. PeerJ.

[19] Fu H., Tan W., Chen Z., Ye Z., Duan Y., Huang J., Qi H., Liu X. (2022). TOP2A deficit-induced abnormal decidualization leads to recurrent implantation failure via the NF-kappaB signaling pathway. Reprod. Biol. Endocrinol..

[20] Yang J., Wang L., Ma J., Diao L., Chen J., Cheng Y., Yang J., Li L. (2023). Endometrial proteomic profile of patients with repeated implantation failure. Front. Endocrinol..

[21] Zhao J., Hao J., Xu B., Li Y. (2023). Recurrent implantation failure versus recurrent implantation success: A preliminary study at proteomic level. Gynecol. Endocrinol..

[22] Perez-Deben S., Bellver J., Alama P., Salsano S., Quinonero A., Sebastian-Leon P., Diaz-Gimeno P., Dominguez F. (2019). iTRAQ comparison of proteomic profiles of endometrial receptivity. J. Proteom..

[23] Inagaki N., Stern C., McBain J., Lopata A., Kornman L., Wilkinson D. (2003). Analysis of intra-uterine cytokine concentration and matrix-metalloproteinase activity in women with recurrent failed embryo transfer. Hum. Reprod..

[24] Yoshii N., Hamatani T., Inagaki N., Hosaka T., Inoue O., Yamada M., Machiya R., Yoshimura Y., Odawara Y. (2013). Successful implantation after reducing matrix metalloproteinase activity in the uterine cavity. Reprod. Biol. Endocrinol..

[25] Benkhalifa M., Zayani Y., Bach V., Copin H., Feki M., Benkhalifa M., Allal-Elasmi M. (2018). Does the dysregulation of matrix metalloproteinases contribute to recurrent implantation failure?. Expert. Rev. Proteom..

[26] Azkargorta M., Escobes I., Iloro I., Osinalde N., Corral B., Ibanez-Perez J., Exposito A., Prieto B., Elortza F., Matorras R. (2018). Differential proteomic analysis of endometrial fluid suggests increased inflammation and impaired glucose metabolism in non-implantative IVF cycles and pinpoints PYGB as a putative implantation marker. Hum. Reprod..

[27] Kasvandik S., Saarma M., Kaart T., Rooda I., Velthut-Meikas A., Ehrenberg A., Gemzell K., Lalitkumar P.G., Salumets A., Peters M. (2020). Uterine Fluid Proteins for Minimally Invasive Assessment of Endometrial Receptivity. J. Clin. Endocrinol. Metab..

[28] Liu Z., Liu X., Wang M., Zhao H., He S., Lai S., Qu Q., Wang X., Zhao D., Bao H. (2022). The Clinical Efficacy of Personalized Embryo Transfer Guided by the Endometrial Receptivity Array/Analysis on IVF/ICSI Outcomes: A Systematic Review and Meta-Analysis. Front. Physiol..

[29] Cozzolino M., Diaz-Gimeno P., Pellicer A., Garrido N. (2022). Use of the endometrial receptivity array to guide personalized embryo transfer after a failed transfer attempt was associated with a lower cumulative and per transfer live birth rate during donor and autologous cycles. Fertil. Steril..

[30] Ahrens C.H., Brunner E., Qeli E., Basler K., Aebersold R. (2010). Generating and navigating proteome maps using mass spectrometry. Nat. Rev. Mol. Cell Biol..

[31] Riesewijk A., Martin J., van Os R., Horcajadas J.A., Polman J., Pellicer A., Mosselman S., Simon C. (2003). Gene expression profiling of human endometrial receptivity on days LH+2 versus LH+7 by microarray technology. Mol. Hum. Reprod..

[32] Hu S., Yao G., Wang Y., Xu H., Ji X., He Y., Zhu Q., Chen Z., Sun Y. (2014). Transcriptomic changes during the pre-receptive to receptive transition in human endometrium detected by RNA-Seq. J. Clin. Endocrinol. Metab..

[33] Hromadova L., Tokareva I., Vesela K., Travnik P., Vesely J. (2019). Endometrial Receptivity Analysis—A tool to increase an implantation rate in assisted reproduction. Ces. Gynekol..

[34] Diaz-Gimeno P., Ruiz-Alonso M., Blesa D., Bosch N., Martinez-Conejero J.A., Alama P., Garrido N., Pellicer A., Simon C. (2013). The accuracy and reproducibility of the endometrial receptivity array is superior to histology as a diagnostic method for endometrial receptivity. Fertil. Steril..

[35] Aebersold R., Mann M. (2016). Mass-spectrometric exploration of proteome structure and function. Nature.

[36] Chen J., Schwarz E. (2017). Opportunities and Challenges of Multiplex Assays: A Machine Learning Perspective. Methods Mol. Biol..

[37] Altmae S., Esteban F.J., Stavreus-Evers A., Simon C., Giudice L., Lessey B.A., Horcajadas J.A., Macklon N.S., D’Hooghe T., Campoy C. (2014). Guidelines for the design, analysis and interpretation of ‘omics’ data: Focus on human endometrium. Hum. Reprod. Update.

